# Modified Endoscopic Submucosal Dissection—An Alternative Modality for the Treatment of Sporadic Duodenal Papillary Adenomas

**DOI:** 10.1155/2024/7444677

**Published:** 2024-10-17

**Authors:** Zelong Han, Qingyuan Li, Chanelle Yeh Chua, Zhenjiang Wang, Jun Zhong, Ying Huang, Shaohui Huang, Aimin Li, Side Liu, Xiaobei Luo

**Affiliations:** ^1^Department of Gastroenterology, Guangdong Provincial Key Laboratory of Gastroenterology, Nanfang Hospital, Southern Medical University 510515, Guangzhou, China; ^2^Department of Internal Medicine, Medical College of Wisconsin, Milwaukee 53235, Wisconsin, USA; ^3^Department of Gastroenterology, Zhuhai People's Hospital (Zhuhai Hospital Affiliated With Jinan University) 519000, Zhuhai, China; ^4^Pazhou Lab 510005, Guangzhou, China

## Abstract

**Background and Aim:** Endoscopic submucosal dissection (ESD) is commonly employed in the treatment of epithelial gastrointestinal tumors, but few studies have explored ESD for treatment of duodenal papillary adenomas (PAs). In this study, we aim to evaluate the feasibility of a modified ESD method as an alternative modality in the resection of PAs.

**Methods:** We evaluated potential advantages of modified ESD for resection of sporadic duodenal PAs through retrospective analysis of 10 PAs resected via ESD compared to paired lesions undergoing endoscopic papillectomy (EP).

**Results:** All lesions undergoing ESD were resected en bloc with negative margins, compared to 60% of lesions undergoing EP. Within the experimental group, there was one case each of melena and pancreatitis compared to four bleeds and one case of pancreatitis in the control group. No recurrence was detected within the ESD group after a mean follow-up time of 11.2 months compared to three recurrences within a mean follow-up time of 27.7 months.

**Conclusions:** From our preliminary experience, ESD is a promising alternative in the treatment of superficial duodenal PAs; however, further investigation is needed.

## 1. Introduction

Duodenal papillary adenomas (PAs) are premalignant neoplasms of the ampulla of Vater. Surgical resection is essential for the prevention of disease progression [1]. Previously, PAs frequently went undetected until progression to malignancy, but as recognition of the therapeutic value of endoscopy has grown, so too has detection of incidental PAs [2]. Common therapeutic approaches include surgical resection, pancreatoduodenectomy, and endoscopic papillectomy (EP). Although EP circumvents some of the morbidity of surgery, limitations include risk of incomplete removal, local recurrence, and complications including intraoperative and postoperative bleeding [3]. While lesions with intraductal extension > 10 mm, friability, or ulceration should be preferentially considered for surgical resection, there is no consensus regarding a size threshold above which EP should not be attempted [1]. As freehand dissection techniques accommodate irregular lesions, endoscopic submucosal dissection (ESD) is increasingly utilized for gastrointestinal epithelial tumors due to high en bloc resection rates. Nevertheless, few studies have explored application of ESD to PAs. Here, we examine the feasibility of a modified ESD method as an alternative modality in the resection of PAs.

## 2. Materials and Methods

This study was a single-center retrospective observational study. We retrospectively collected data from 10 patients with superficial PAs who underwent modified ESD from July 2019 to May 2021 at Nanfang Hospital, Guangzhou, China. Data from 10 paired lesions undergoing EP from December 2013 to March 2021 was also collected retrospectively to further evaluate the potential advantages and disadvantages of ESD compared to EP. The procedures were performed by two experienced endoscopists (ESD > 200 cases). The study protocol (NFEC-2017-164) was approved by the Institutional Review Board at Nanfang Hospital.

Endoscopic assessment with biopsy, endoscopic ultrasonography (EUS), and upper abdominal CT or MRI were performed prior to ESD and EP. Lesions with macroscopic signs of ulceration, friability, or evidence of intraductal extension or muscularis propria involvement were excluded. Patients in both study groups underwent routine endoscopic surveillance.

### 2.1. ESD Procedure (Supporting Information 1 and 2: Video Clips S1 and S2)

**Figure 1:**
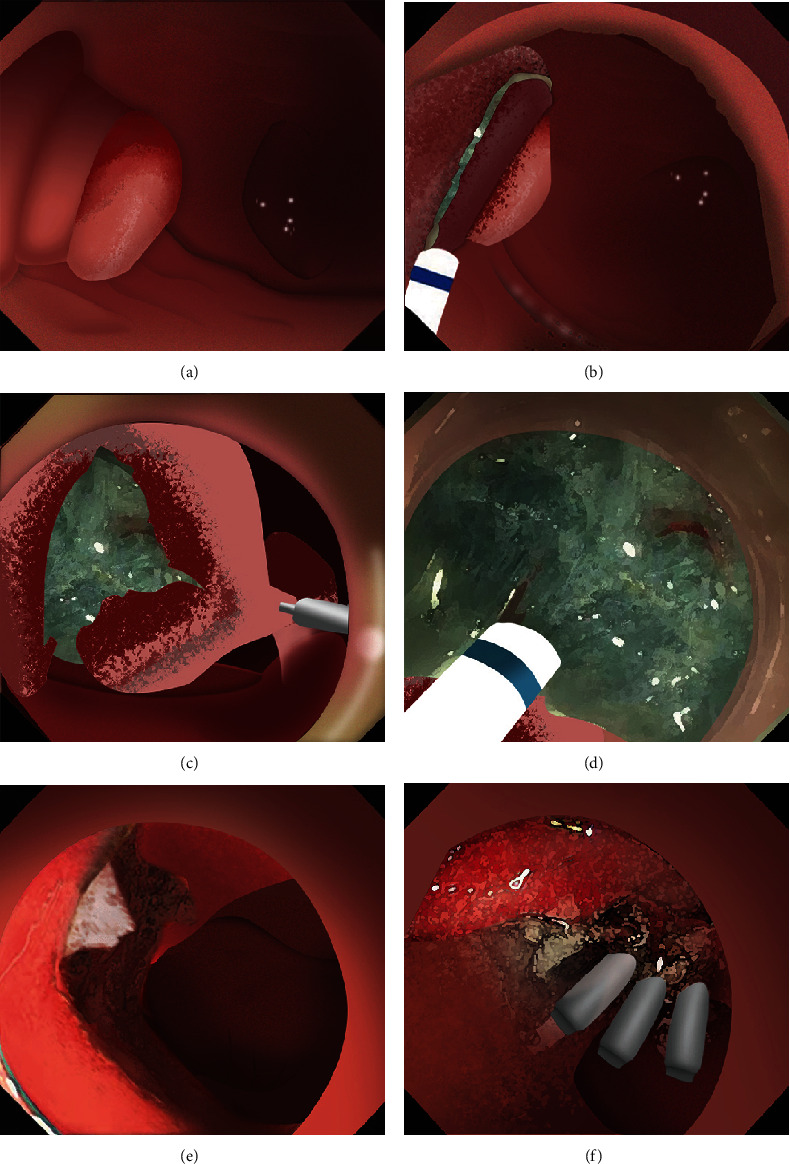
The modified ESD technique for the treatment of superficial PAs. (a) A major papillary adenoma. (b) Submucosal injection and a semicircular mucosal incision. (c) Traction-assisted endoscopic submucosal dissection. (d) Submucosal layer was dissected until the lesion was completely resected. (e) The wound. (f) The wound was partially closed.

Single-channel esophagogastroduodenoscopes (EGD, GIF-Q260J, Olympus, Japan) were fitted with transparent caps for the operation. Procedure steps are illustrated in Figure 1. Submucosal dissection was performed using “EndoCut I” modality (effect 3, cutting duration 3) (ERBE, Tubingen, Germany). Soft coagulation (80 W on effect 4) was used for hemostasis.

### 2.2. Mucosal Incision

Submucosal injection of saline solution and indigo carmine was performed proximal to the lesion. A semicircular mucosal incision was made before submucosal dissection with a DualKnife J (KD655L, Olympus, Japan) or a HybridKnife (I type, ERBE, Germany). Buscopan was administered for intensive intestine peristalsis.

### 2.3. Submucosal Dissection

Dissected mucosal flaps were anchored to the contralateral intestinal wall using endoclips. Internal traction enabled adequate submucosal exposure in the duodenal lumen and facilitated visualization of submucosal vessels. Large submucosal vessels were coagulated to minimize bleeding. During the dissection, the sphincter of Oddi is visualized as a muscular complex in the submucosal plane. It is essential to dissect this muscular complex carefully, maintaining a dissection line parallel to the exposed submucosal plane. This approach is necessary to avoid shallow dissection and prevent potential perforation. The submucosal layer was dissected towards the distal part of the lesion until complete resection.

### 2.4. Prophylactic Clip Closure

Exposed vessels on the raw surface were soft-coagulated, and mucosal defects distal to the papilla were closed with endoclips with or without endoloops. Proton pump inhibitors, somatostatin, or rectal NSAIDs were administered perioperatively. Serum amylase and lipase were monitored postoperatively.

## 3. Results

**Figure 2:**
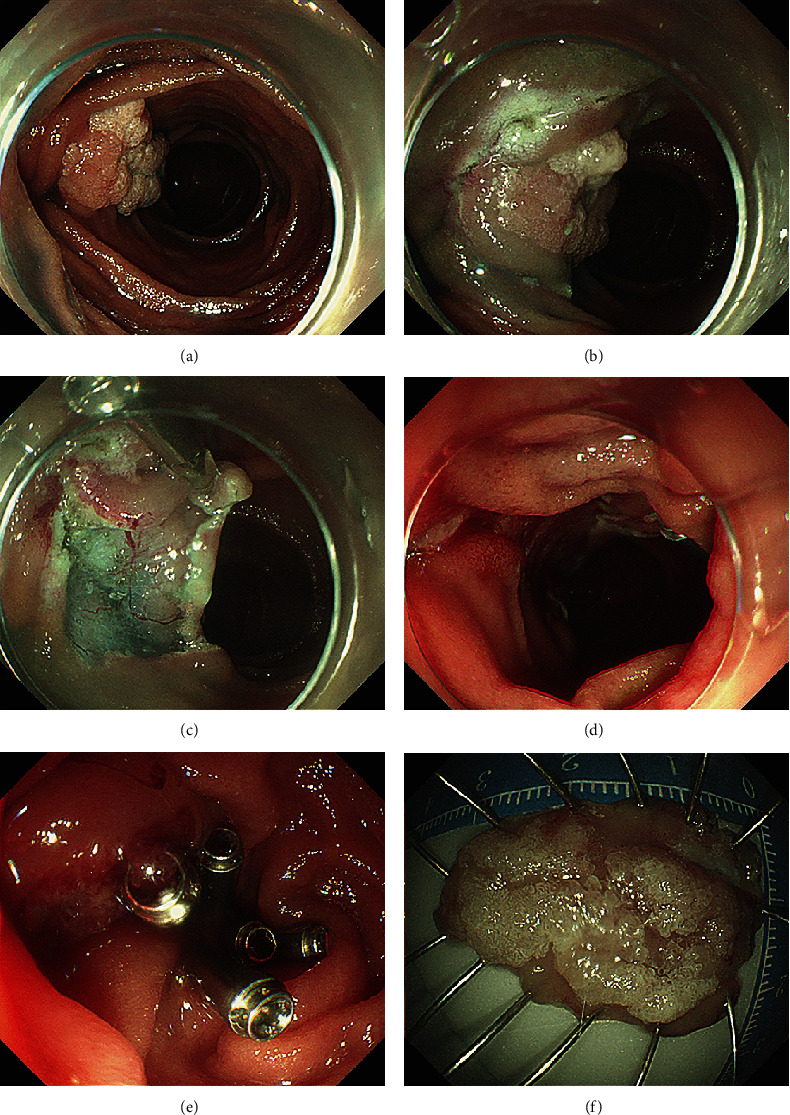
A LST located on the major papilla resected by the modified ESD technique. (a) A 35 mm × 25 mm LST located on the papilla. (b) A mucosal incision is made over the semicircle. (c) Internal traction made using an endoclip. (d) Submucosal dissection was performed until the lesion was resected completely. (e) Wound partially closed with clips. (f) The resected specimen.

Ten patients (mean age 51.6 years, SD 16.8 years, 7 males) with PAs underwent ESD. ESD was technically successful in six laterally spreading tumors (LSTs) (mean size 32.5 mm, range 25–45 mm) and four ampullary adenomas (AAs) (mean size 15.5 mm, range 12–23 mm). Characteristics of the patients and lesions are summarized in Table 1. All lesions were resected en bloc (Figure 2, Table 1, and Supporting Information 3: Figure S1) with negative margins. The average specimen size was 25.7 mm (SD 10.78 mm, range 12–45 mm). Pathological examination of the resected specimens revealed low-grade dysplasia (LGD) in five patients, high-grade dysplasia (HGD) in four patients, and HGD with intramucosal adenocarcinoma in one patient. The average operation time was 50 min (SD 15.97 min, range from 25 to 80 min). No major bleeding or any perforation occurred during the operations. One patient experienced an episode of melena following resection of a LST, and one patient displayed elevated amylase and lipase. However, both patients were discharged after conservative treatment without additional procedural intervention. No perforation, stenosis, cholangitis, or deaths occurred, and no recurrence was detected after a mean endoscopic follow-up time of 11.2 (range 3–29) months.

The patients undergoing ESD were paired with ten patients (mean age 53.9 years, SD 12.8 years, 7 males) undergoing EP. Lesions were matched for size and pathology (Table 2). EP was performed on six LSTs (mean size 34.2 mm, range 30–45 mm) and four AAs (mean size 17.25, range 13–20 mm). Four lesions including three LSTs were resected in a piecemeal fashion (Table 2). Three patients experienced postoperative bleeding, and one experienced postoperative pancreatitis. One LST that underwent piecemeal resection demonstrated recurrence 1 month postprocedure, and three lesions displayed recurrence during a mean follow up period of 27.7 (range 1–68) months.

## 4. Discussion

ESD is well established in the treatment of superficial gastrointestinal neoplasms with higher en bloc resection rates compared to snare resection and endoscopic mucosal resection (EMR). However, ESD has thus far been rarely used in the treatment of PAs due to technical difficulties involved in performing ESD in the descending part of the duodenum (D2) and around the duodenal papilla [4]. In this study, we achieved a 100% success rate in the removal of superficial PAs. ESD procedures were modified to suit the anatomy of the duodenum. We postulate that clip traction optimizes tissue tension within the submucosa and exposure of the dissection line. Direct visualization of the submucosal vessels facilitates pretreatment of submucosal vessels in the ampullary region and minimizes major intraoperative bleeding.

Although some studies suggest prophylactic pancreatic duct (PD) stent placement reduces the risk of postprocedural pancreatitis in EP [5], routine PD stent placement is not always successful and has been questioned in recent case series [6, 7]. The benefit of routine deployment of PD stents following ESD is unclear at this time. In lieu of prophylactic biliary or pancreatic stents, somatostatin or rectal NSAIDs were administered perioperatively. One patient with mild pancreatitis was observed postoperatively, but the patient was asymptomatic, and serum biomarkers decreased to less than 3 times the upper limit of normal after 48 h of conservative treatment. None of the patients undergoing ESD had postoperative complications requiring further procedural intervention, and the rate of postoperative pancreatitis was comparable to that of EP described with PD stent placement (7.2%–20%) [8, 9]. However, given the sample size, further studies with more cases are needed to evaluate ideal postoperative management and adverse events in the removal of PAs using this technique.

Currently, EP is favored over surgical resection for the treatment of superficial PAs due to its minimally invasive approach [7]. Reported rates of en bloc resection and local recurrence using snare resection or EMR for AA and ampullary LST range from 59.1% to 90.3% and 5.0% to 22.9% [9–13], respectively, and the recurrence rate was reported to be 8%–33% [3, 10, 14]. Studies have shown that histologically confirmed recurrence is associated with piecemeal resection (*p* = 0.02) and number of pieces (*p* = 0.02) [3]. One of the most important advantages of ESD is that it enables a high rate of en bloc resection and thus significantly reduces the rates of incomplete tumor resection, especially for large, irregular lesions. Intact specimens following ESD facilitate accurate histopathological assessment. A R0 resection rate of 100% was achieved in the ESD group, and no recurrence was detected with a mean of 11.2 months compared to a 30% recurrence rate in lesions that underwent EP with a mean of 27.7 months. Of note, EP was performed prior to ESD, and as such, the mean follow-up period was shorter in the ESD group. Even so, our sample supports the hypothesis that ESD results in a reduction of local recurrence compared to EP.

To our knowledge, this is the first case series exploring the application of ESD techniques in PAs. The high en bloc resection rate and low adverse event rate of the lesions in the study group as compared to the control suggest that this modified ESD procedure provides an alternative endoscopic approach to EP for the treatment of superficial PAs, particularly for LSTs. Nevertheless, as ESD procedures within D2 are more technically challenging than those in the stomach or colon, ESD in PAs should be performed only by endoscopists with extensive ESD experience.

This is a single-center retrospective observational study with limited sample size and follow-up time. Prospective controlled trials with a larger number of cases are needed to examine the advantages of higher en bloc resection rate and decreased recurrence as compared to traditional approaches, to evaluate rates of adverse events throughout time, and to determine ideal postoperative management. Additional comparison of ESD and EP approaches to PA resection is needed to explore the optimal indications for these minimally invasive treatment strategies.

## 5. Conclusions

Our data supports ESD as a promising alternative in the treatment of superficial duodenal PAs. This study provides a foundation for further investigation supporting the application of ESD techniques towards PAs and suggests that ESD techniques may be particularly helpful in reducing recurrence in LSTs with similar complication rates as EP.

## Figures and Tables

**Table 1:** Clinical characteristics of the 10 consecutive cases of the major papillary tumors that have been successfully resected by this modified ESD technique.

**Patient no.**	**Gender**	**Age**	**Tumor size (mm)**	**LST**	**Operating time (min)**	**Estimated blood loss (mL)**	**Pathology**	**En bloc resection**	**Intraoperative or postoperative perforation**	**Postoperative bleeding**	**Postoperative pancreatitis**	**Follow-up (month)**
1	Male	70	15 × 15	None	50	≈0	Low-grade	Yes	None	None	Mild	29
2	Male	32	23 × 15	None	60	1	Low-grade	Yes	None	None	None	18
3	Female	58	35 × 25	Yes	40	2	High-grade	Yes	None	None	None	16
4	Male	70	12 × 12	None	50	1	Low-grade	Yes	None	None	None	3
5	Male	41	25 × 20	Yes	25	≈0	High-grade	Yes	None	None	None	14
6	Female	44	40 × 40	Yes	60	≈0	High-grade	Yes	None	None	None	6
7	Male	73	12 × 12	None	60	1	High-grade	Yes	None	None	None	6
8	Male	53	23 × 25	Yes	80	1	High-grade, intramucosal cancer	Yes	None	Yes	None	9
9	Male	19	25 × 20	Yes	25	1	Low-grade	Yes	None	None	None	8
10	Female	56	45 × 25	Yes	50	≈0	Low-grade	Yes	None	None	None	3

**Table 2:** Clinical characteristics of the 10 paired papillary tumors resected by the endoscopic papillectomy.

**Patient no.**	**Gender**	**Age**	**Tumor size (mm)**	**LST**	**Pathology**	**En bloc resection**	**Intraoperative or postoperative perforation**	**Postoperative bleeding**	**Postoperative pancreatitis**	**Follow-up (month)**
1	Male	69	20 × 10	No	Low-grade	No	None	Yes	None	68
2	Male	55	20 × 15	No	Low-grade	Yes	None	Yes	None	25
3	Male	49	35 × 25	Yes	High-grade	No	None	None	None	57
4	Female	59	16 × 8	No	Low-grade	Yes	None	None	None	45
5	Female	77	30 × 20	Yes	High-grade	No	None	None	None	18
6	Male	54	35 × 35	Yes	High-grade	Yes	None	Yes	None	37
7	Male	51	13 × 12	None	High-grade	Yes	None	None	None	61
8	Female	47	30 × 20	Yes	High-grade	Yes	None	Yes	None	3
9	Male	26	30 × 20	Yes	Low-grade	Yes	None	None	Yes	39
10	Female	52	45 × 25	Yes	Low-grade	No	None	None	None	1

## Data Availability

The data that support the findings of this study are available in the Supporting Information section.

## Supporting Information

Supporting Information 1 Video Clip S1. The video shows the en bloc resection of a duodenal PA using the modified ESD technique. The operating time was approximately 60 min. No major bleeding and perforation occurred during the procedure.

Supporting Information 2 Video Clip S2. The video shows the resection of a 45 mm × 25 mm LST located on the major papilla, and the pathological results revealed a tubulovillous adenoma with focal high-grade intraepithelial neoplasia. The operation required approximately 50 min. No postoperative complications were noted.

Supporting Information 3 Figure S1. A superficial major papillary adenoma resected by the modified ESD technique. (a) A superficial papillary adenoma. (b) Submucosal injection using a DualKnife J. (c) A semicircular mucosal incision and traction-assisted endoscopic submucosal dissection. (d) The wound. (e) The wound was partially closed. (f) The resected specimen.
