# Supplementary material for: Modified Endoscopic Submucosal Dissection—An Alternative Modality for the Treatment of Sporadic Duodenal Papillary Adenomas
Source: Gastroenterol Res Pract. 2024 Oct 17;2024:7444677. doi: 10.1155/2024/7444677 (PMC11502125; doi:10.1155/2024/7444677)
Supplement: Supporting Information 1 — Video Clip S1. The video shows the en bloc resection of a duodenal PA using the modified ESD technique. The operating time was approximately 60 min. No major bleeding and perforation occurred during the procedure. [file 7444677.f1.doc]

<https://drive.google.com/file/d/17WVNS1VmdaFsgv9_Vx1Cc9TyfFCbM5vn/view?usp=drivesdk>
