# Supplementary material for: Modified Endoscopic Submucosal Dissection—An Alternative Modality for the Treatment of Sporadic Duodenal Papillary Adenomas
Source: Gastroenterol Res Pract. 2024 Oct 17;2024:7444677. doi: 10.1155/2024/7444677 (PMC11502125; doi:10.1155/2024/7444677)
Supplement: Supporting Information 2 — Video Clip S2. The video shows the resection of a 45 mm × 25 mm LST located on the major papilla, and the pathological results revealed a tubulovillous adenoma with focal high-grade intraepithelial neoplasia. The operation required approximately 50 min. No postoperative complications were noted. [file 7444677.f2.doc]

<https://drive.google.com/file/d/1s1cD9ijMxChe9pAtRCSw46DbWDApqvm6/view?usp=drivesdk>
