# Supplementary figures and images for: Modified Endoscopic Submucosal Dissection—An Alternative Modality for the Treatment of Sporadic Duodenal Papillary Adenomas
Source: Gastroenterol Res Pract. 2024 Oct 17;2024:7444677. doi: 10.1155/2024/7444677 (PMC11502125; doi:10.1155/2024/7444677)

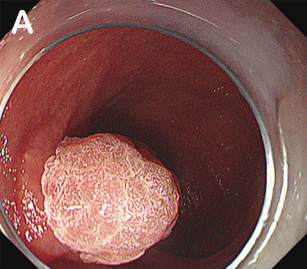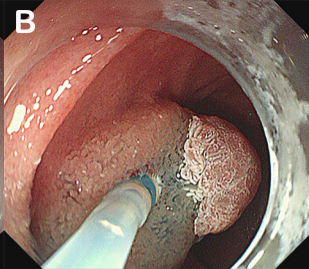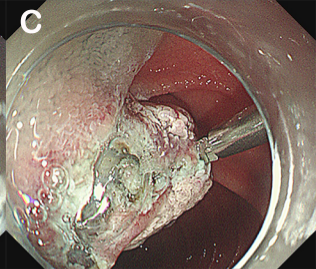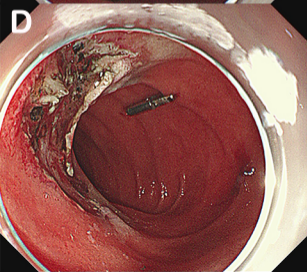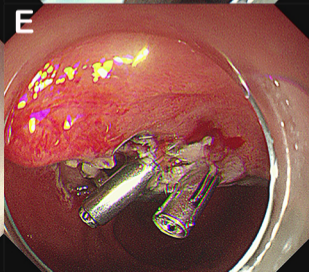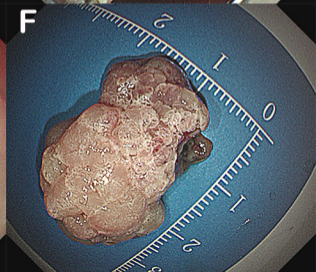

Supplement: Supporting Information 3 — Figure S1. A superficial major papillary adenoma resected by the modified ESD technique. (a) A superficial papillary adenoma. (b) Submucosal injection using a DualKnife J. (c) A semicircular mucosal incision and traction-assisted endoscopic submucosal dissection. (d) The wound. (e) The wound was partially closed. (f) The resected specimen. [file 7444677.f3.pdf]
